# The Adoption of a Virtual Reality–Assisted Training System for Mental Rotation: A Partial Least Squares Structural Equation Modeling Approach

**DOI:** 10.2196/14548

**Published:** 2020-01-17

**Authors:** Chen-Wei Chang, Shih-Ching Yeh, Mengtong Li

**Affiliations:** 1 School of Journalism Fudan University Shanghai China; 2 Department of Computer Science & Information Engineering National Central University Taoyuan City Taiwan; 3 Department of Psychology Fudan University Shanghai China

**Keywords:** virtual reality, computer simulation, educational technology, training programs

## Abstract

**Background:**

Virtual reality (VR) technologies have been developed to assist education and training. Although recent research suggested that the application of VR led to effective learning and training outcomes, investigations concerning the acceptance of these VR systems are needed to better urge learners and trainees to be active adopters.

**Objective:**

This study aimed to create a theoretical model to examine how determining factors from relevant theories of technology acceptance can be used to explain the acceptance of a novel VR-assisted mental rotation (MR) training system created by our research team to better understand how to encourage learners to use VR technology to enhance their spatial ability.

**Methods:**

Stereo and interactive MR tasks based on Shepard and Metzler’s pencil and paper test for MR ability were created. The participants completed a set of MR tasks using 3D glasses and stereoscopic display and a 6-degree-of-freedom joystick controller. Following task completion, psychometric constructs from theories and previous studies (ie, perceived ease of use, perceived enjoyment, attitude, satisfaction, and behavioral intention to use the system) were used to measure relevant factors influencing behavior intentions.

**Results:**

The statistical technique of partial least squares structural equation modeling was applied to analyze the data. The model explained 47.7% of the novel, VR-assisted MR training system’s adoption intention, which suggests that the model has moderate explanatory power. Direct and indirect effects were also interpreted.

**Conclusions:**

The findings of this study have both theoretical and practical importance not only for MR training but also for other VR-assisted education. The results can extend current theories from the context of information systems to educational and training technology, specifically for the use of VR-assisted systems and devices. The empirical evidence has practical implications for educators, technology developers, and policy makers regarding MR training.

## Introduction

### Background

Research on the acceptance of new information systems and technologies provides empirical evidence guiding decision-making processes in regard to system developments, educational implications, and other practices. For example, in the fields of information systems and behavioral science, robust models (eg, technology acceptance model, TAM; [1]) have been proposed and repeatedly replicated in different contexts to examine and better understand the processes of accepting new technologies and systems. In recent years, virtual simulation tools such as the VIVE (HTC Corporation) have been developed and have become more prevalent. These tools create a user experience that better engages cognitive, visual, and motional perceptions. As virtual reality (VR) is different from other information systems—in that users can perceive stereoscopic 3D objects and it is capable of changing fields-of-view while simultaneously interacting with the content via virtual controllers or motion sensors—this study sees a need to explore users’ acceptance of such emergent technology.

Although VR technologies are relatively new and require acceptance research, they have been applied to modern learning and training systems and shown effective outcomes (eg, [2,3]). For instance, a recent study conducted by Yeh et al [2] explored using VR for mental rotation (MR) training. MR is a kind of cognitive ability that uses human spatial imagination to rotate an object in a 2D or 3D space [2,4,5]. In a stereoscopic and interactive virtual environment, the results of pretest-posttest comparison suggested that the VR training improved learners’ MR ability [2]. These results led to this study’s interest in gauging the factors that would encourage learners to use the VR system to enhance their spatial ability, as the MR ability influences an individual’s learning activities relying on spatial cognitions including those in the field of medical science. Take anatomy learning as an example. Research has shown that MR ability and the outcome of anatomy learning were positively correlated [6,7]. Hoyek et al [7] conducted an experiment comparing 3 groups of anatomy learning: (1) attending anatomy class and receiving MR training (ie, the treatment group), (2) attending anatomy class only (ie, the comparison group), and (3) neither attending anatomy class nor receiving MR training (ie, the control group). The results concluded that the intervention of MR training positively influenced the students’ performance in the anatomy test.

Given the developments and research stated above, this study intended to understand the users’ complex psychological processes of accepting the VR-assisted MR training system. The empirical results could extend current theories of acceptance from the context of information systems to educational and training technology, specifically for VR-assisted systems and devices. The main goals of this study were, therefore, to (1) create a behavioral model to better understand the adoption of VR technology for MR training, (2) provide suggestions to educators and technology designers regarding the application and improvement of VR training technology for MR, and (3) establish a stepping stone for future researchers and practitioners interested in incorporating VR technology into other educational and training activities for their users.

### Theoretical Framework and Research Hypotheses and Question

To comprehend how an information system is accepted by users, Davis et al [1] proposed the TAM, developed from Fishbein and Ajzen’s [8] Theory of Reasoned Action for predicting and understanding an individual’s rational behavior and decision making. The TAM model has been frequently used to research the acceptance of a new information and computer system and technology, including those for educational purposes and settings (eg, [9,10]) and has recently been applied to better understand the adoption of VR-assisted training and educational systems (eg, [11,12]). Regarding the design of user interface, TAM assumes that perceived ease of use (PEOU) influences users’ attitude (ATT) toward an information system and that ATT affects their behavioral intention to use (BIU) it. This study hypothesized that the same effects apply to our VR-assisted MR training system.

H1: Users’ PEOU of the MR system predicts their positive ATT toward it.

H2: Users’ positive ATT toward the system predicts their BIU.

As technology has developed, the TAM model has progressed and evolved. Davis et al [13] found that both extrinsic (eg, perceived usefulness [PU] and PEOU) and intrinsic (eg, cognitive enjoyment) motivations affected users’ behavioral intention and actual use of an information system. Extrinsic motivations are involved with the rewards via actual use, such as the enhancement of job efficiency and increase of salary. On the other hand, intrinsic motivations emphasize the rewards in the experience as the ultimate goal, such as the enjoyment of an activity. By empirically testing the relationships of intrinsic and extrinsic motivations, Davies found that PU and perceived enjoyment (PE) were affected by PEOU. Hence, we postulated H3 for the use of our system.

H3: Users’ PEOU affects their PE when using the MR training system.

Except for the PU and PEOU as the 2 determinants, researchers started to explore an individual’s enjoyable experiences after Davis et al [13]. PE has been verified to have effects on ATT and satisfaction (SAT) with information systems and technology. For example, PE influenced users’ positive ATT and intention to use the World Wide Web [14]. In digital learning, trainees’ cognitive playfulness affected their learning outcomes, positive mood, and SAT with the training system [15]. On the basis of this evidence, we proposed H4 and H5.

H4: Users’ PE predicts their positive ATT toward the MR training system.

H5: Users’ PE predicts their SAT with the MR training system.

Previous studies suggested that users’ SAT is a predictive factor for the adoption of new information technology. According to expectation-confirmation model (ECM; [16]), users’ perception of usefulness and confirmation (ie, whether the experience of information technologies meets their expectation) influences their SAT with the system, and the level of SAT predicts their continued intention to use the system. Ho’s [17] study on electronic learning platforms found that users’ SAT with the technology also affected their ATT toward the system. Given these findings from previous relevant studies, we hypothesized that trainees’ SAT with the VR-assisted MR training system would influence both their ATT toward and intention to use the system.

H6: Users’ SAT with the system predicts their positive ATT.

H7: Users’ SAT with the system predicts their BIU.

**Figure 1:**
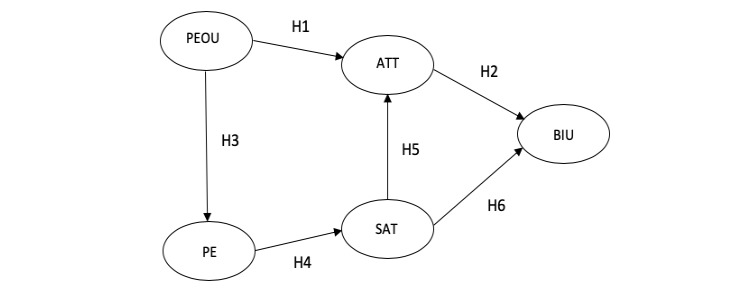
The proposed theoretical research model includes perceived ease-of-use (PEOU), perceived enjoyment (PE), attitude (ATT), satisfaction (SAT), and behavioral intention to use the system (BIU). H: hypothesis.

Figure 1 shows all the proposed hypotheses and the research model for this study.

To better understand how each factor interacts with others to affect learners’ intention to use the VR-assisted MR training system and to provide more detailed implications for future researchers and practitioners, we asked the following research question to gauge the indirect effects among the constructs in this study.

RQ: Are there any indirect effects among the constructs in the proposed model?

## Methods

### System Design

**Figure 2:**
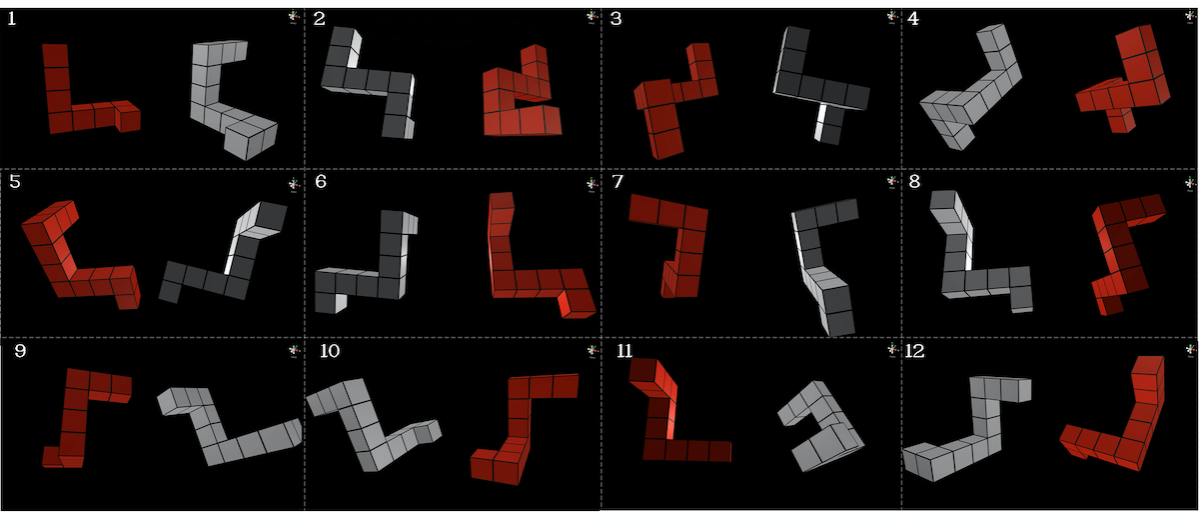
The stereoscopic interactive mental rotation tasks created by the research team.

**Figure 3:**
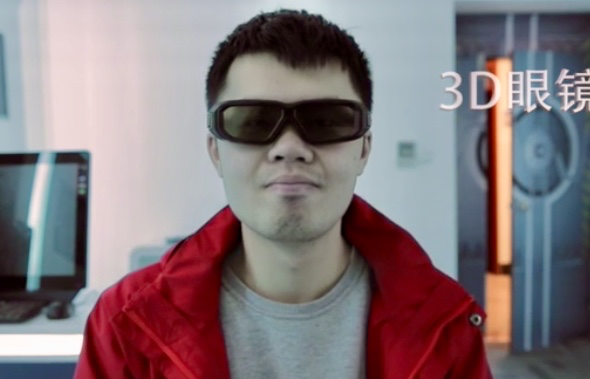
3D glasses were used to facilitate the interactive virtual reality experience for mental rotation tasks (written, informed consent was obtained from the individual for publication of the image).

**Figure 4:**
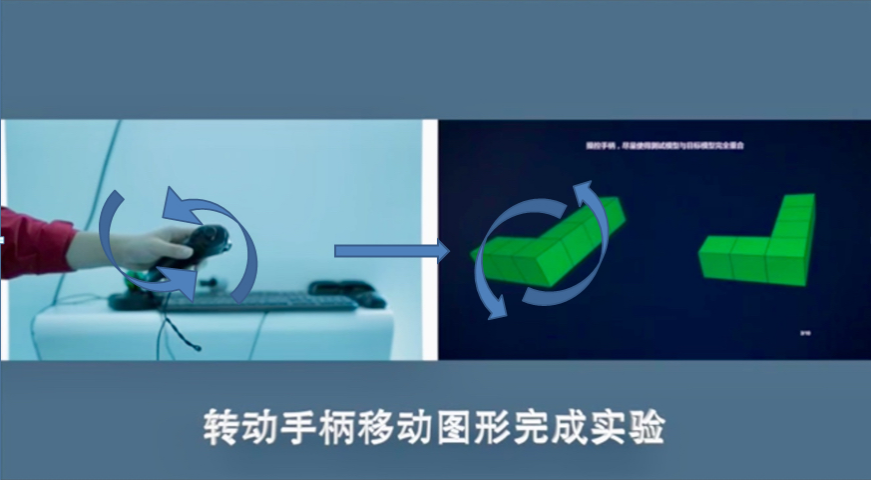
Joystick controller was used to facilitate the interactive virtual reality experience for mental rotation tasks.

Shepard and Metzler [18] developed a paper-based MR test. Two 3D objects having the same geometric form but in different orientations were created on a 2D sheet. Trainees were typically asked to identify whether these 2 objects are the same. Developed from Shepard and Metzler’s MR tasks, this study adopted 12 stereo and interactive MR tasks (2 for the purpose of practice and 10 for formal training) for our VR-assisted interactive MR training system. OpenGL (Khronos Group) [19], a computer graphic software, was used to create the 3D objects (see Figure 2 for the visual demonstration), and Quad-Buffer Stereo [20] was used in OpenGL to create 120 frames per second for stereovision. For the hardware, RealID shutter glasses (ie, 3D glasses) were used by the participants (Figure 3). The mechanism of stereovision relies on visual persistence and the cross-display of 120 images between 2 eyes. For the interactive experience, Unity engine [21] was applied, so users could virtually rotate the MR subjects via a 6-degree-of-freedom (dof) controller (ie, joystick; Figure 4). In our study, trainees were asked to observe and rotate an MR object and drag it to superimpose a replicate on the other side of the screen that has the same geometric form but a different orientation. Once the 2 MR objects perfectly matched, the specific single MR task was accomplished.

### Participants and Procedure

This study used partial least squares structural equation modeling (PLS-SEM) for data analysis. PLS-SEM is one of the most used analytical technique by management information system and marketing researchers, and studies applying PLS-SEM have been published in journals such as *MIS Quarterly* [22]. PLS-SEM suggests a 10-time rule for the sample size. The sample size must be 10 times greater than (1) the maximum number of paths a latent variable has and (2) the greatest indicators (items) a latent variable has in the model [23]. This study used a data subset of 55 participants from an earlier developed experimental study [24], which surpassed the 2 threshold requirements. The students in this study were all recruited from the same large research university in Shanghai, China, in early 2018. Each of them received 10 RMB (about US $1.5) to compensate their time. Their ages ranged from 18 to 26 years (mean 20.29, SD 1.85; median 20.00), with a control of approximate numbers of male (n=29) and female (n=26) participants. They came from diverse majors, including journalism, social science, psychology, Chinese, German, museum studies, mathematics, science, physics, microelectronics, electrical engineering, medicine, medical, chemistry, and electronical information science and technology. For the conditions analyzed in this study, the participants were first asked to watch an instruction video to understand the basic concept of MR and how to use the VR-assisted training equipment. Afterward, they were asked to use the VR equipment (ie, the stereoscopic display and 3D glasses and joystick controller) to perform 2 MR tasks for practice and 10 MR tasks for formal training, which were created by our research team, followed by a Web-based questionnaire. Owing to the nature of a data subset from a previous experimental study applying a reverse group design, nearly half of the participants in this study had experienced another condition—using a 3-dof computer mouse as the controller before their repeated practice using a 6-dof joystick controller. To prevent potential order effects, we added control variables reflecting the influences caused by the differences between the 2 groups to our analyzed PLS-SEM model. None of the control variable’s effect was statistically significant, which indicated a similarity between groups.

### Measurement

The measurement for the individual constructs were developed from previous theories and research in information systems and technology, some of which were introduced in the literature review (Table 1). Seven-point Likert scales were applied to PEOU, ATT, PE, and BIU. Semantic measures (7-point scales) were used for the constructs of SAT.

**Table 1:** Measurement scales.

Construct	Item	Cronbach alpha
Perceived ease of use [25]	1. My interaction with this MR^a^ system was clear and understandable. 2. Interacting with this MR system did not require a lot of my effort. 3. I found this MR system difficult to use (reversed; withdrawn because of a low factor loading). 4. I found it easy to get this MR system to do what I wanted it to do.	.769
Attitude [26]	1. Using this MR system is a good idea. 2. Using this MR system is a wise idea. 3. I dislike the idea of using this MR system (reversed; withdrawn because of a low factor loading). 4. Using this MR system is pleasant.	.935
Perceived enjoyment [13]	1. I found using this MR system to be enjoyable. 2. The actual process of using this MR system was pleasant. 3. I had fun using this MR system.	.959
Satisfaction [16]	How did you feel about your overall experience of this MR system? (sematic measures): (1) Very dissatisfied <– –> Very satisfied, (2) Very displeased<– –>Very pleased, (3) Very frustrated<– –>Very contented, and (4) Absolutely terrible<– –>Absolutely delighted	.862
Behavioral intention of use [25]	1. Assuming I had access to this MR system, I intend to use it. 2. Given that I had access to this MR system, I predict that I would use it.	.948

^a^MR: mental rotation.

## Results

This study used IBM SPSS and SmartPLS 3 to analyze data. In SmartPLS 3, the *outer model* represents the *measurement model*, whereas the *inner model* is the same as the “structural model” in other structural equation modeling (SEM) software. Before the SEM analysis, the data were examined for outliers and univariate normality distributions. One case was deleted because of its extreme z-score for a measured item. Afterward, the researchers examined the validity and reliability for the outer model in SmartPLS 3. As can be seen in the measurement, all the latent variables were considered internally consistent according to Nunnally and Bernstein’s [27] rule of 0.7 for the Cronbach alpha, as they ranged from 0.769 to 0.959. The convergent validity was met based on 3 criteria: (1) The indicators for individual constructs surpassed the threshold value of 0.7, (2) the composite reliability values were greater than 0.6, and (3) each construct had a coefficient of average variance extracted (AVE) no less than 0.6 ([28,29]; see Table 2 for more details on the convergent validity).

**Table 2:** Convergent validity.

Construct	Item	Standardized loading	Composite reliability	AVE^a^	√AVE
PEOU^b^	PEOU1	0.837	—^c^	—	—
	PEOU2	0.871	—	—	—
	PEOU4	0.772	0.867	0.685	0.828
ATT^d^	ATT1	0.948	—	—	—
	ATT2	0.955	—	—	—
	ATT4	0.920	0.959	0.886	0.941
PE^e^	PE1	0.971	—	—	—
	PE2	0.970	—	—	—
	PE3	0.942	0.973	0.924	0.961
SAT^f^	SAT1	0.793	—	—	—
	SAT2	0.837	—	—	—
	SAT3	0.875	—	—	—
	SAT4	0.853	0.905	0.706	0.840
BIU^g^ the system	BIU1	0.975	—	—	—
	BIU2	0.975	0.975	0.951	0.975

^a^AVE: average variance extracted.

^b^PEOU: perceived ease of use.

^c^Not applicable.

^d^ATT: attitude.

^e^PE: perceived enjoyment.

^f^SAT: satisfaction.

^g^BIU: behavioral intention to use.

For the discriminant validity, the squared root of the AVE value for each construct was compared with the correlations between the construct and other latent variables. The results suggested that the coefficients of √AVE were all greater than other correlations’ Pearson r, which suggests a good discriminant validity for the outer model ([28]; see Table 3). The resulting variance inflation factors also suggested that no significant colinear relationship was identified.

**Table 3:** Correlation coefficients for discriminant validity.

Construct	PEOU^a^	ATT^b^	PE^c^	SAT^d^	BIU^e^
PEOU	0.828	—^f^	—	—	—
ATT	0.521	0.941	—	—	—
PE	0.440	0.808	0.961	—	—
SAT	0.214	0.609	0.618	0.840	—
BIU	0.218	0.634	0.549	0.603	0.975

^a^PEOU: perceived ease of use.

^b^ATT: attitude.

^c^PE: perceived enjoyment.

^d^SAT: satisfaction.

^e^BIU: behavioral intention to use.

^f^Not applicable.

**Figure 5:**
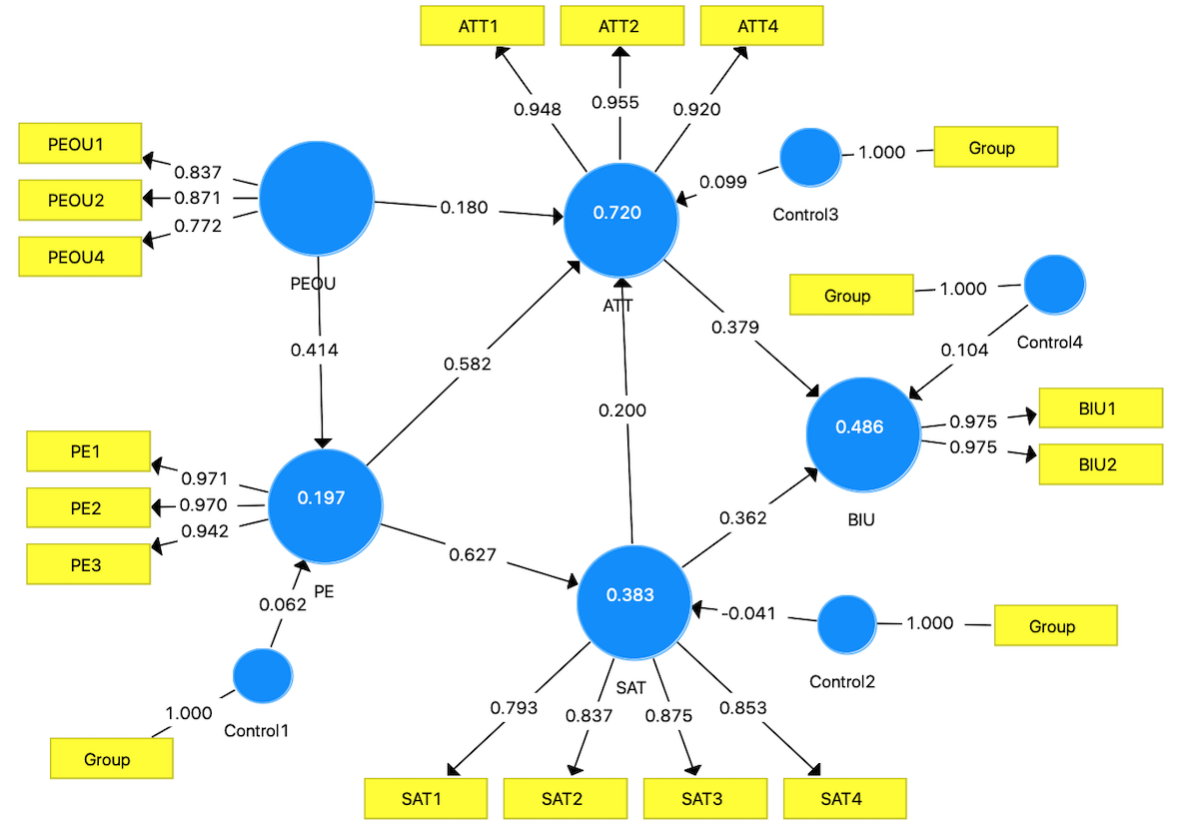
The partial least squares structural equation modeling structural model for the acceptance of our virtual reality–assisted mental rotation training system. ATT: attitude; BIU: behavioral intention to use; PE: perceived enjoyment; PEOU: perceived ease of use; SAT: satisfaction.

Given that the coefficient of standardized root mean squared residual, an index of model fit in SmartPLS 3, was less than .08 (.076 for the structural model and .079 for the estimated model), the data fit the model well [30]. Afterward, the inner model was created applying SmartPLS 3’s PLS algorithm. All the path coefficients can be seen in Figure 5. To test their level of statistical significance, a bootstrapping algorithm was calculated 5000 times in the software, as suggested by Hair et al [31]. The results showed that 4 out of 7 path coefficients were statistically significant at a *P* level of .05. One path was marginally significant at a *P* value of .10 (*P*=.07), and the other 2 paths were close to marginally significant (*P*=.11 and .12; see Table 4). The entire model explained 47.7% of the variance of BIU (including and excluding the control variables for groups for BIU: 48.6% and 47.7%, respectively), which is considered moderately explanatory [32].

**Table 4:** Coefficients for the bootstrapping results.

Path	Original beta (O)	Sampling beta	SD	T (O/SD)	*P* values
ATT^a^–>BIU^b^	.379	.378	0.187	2.028	<.05
PE^c^–>SAT^d^	.627	.634	0.085	7.398	<.001
PE–>ATT	.582	.563	0.141	4.136	<.001
PEOU^e^–>ATT	.180	.185	0.112	1.614	.11
PEOU–>PE	.414	.442	0.141	2.943	<.01
SAT–>ATT	.200	.213	0.128	1.567	.12
SAT–>BIU	.362	.359	0.199	1.819	<.10

^a^ATT: attitude.

^b^BIU: behavioral intention to use.

^c^PE: perceived enjoyment.

^d^SAT: satisfaction.

^e^PEOU: perceived ease of use.

To better understand how each construct interacted with other variables in the model, indirect effects (mediations) were also examined based on a 5000-time bootstrapping sample. As can be seen from Table 5, all indirect effects (including specific paths) for endogenous variables were exported and compared. Regarding the indirect effect on the variable of SAT, PE significantly mediated the influence of PEOU on SAT (beta=.259). For the construct of ATT, the indirect effect via the path PEOU–>PE–>ATT significantly mediated the effect of PEOU on ATT (beta=.241). For BIU, PE and PEOU’s effects were both significantly mediated by other variables (beta=.495 and beta=.273). Specifically, PE’s effect on BIU consisted of the marginally significant paths of PE–>ATT–>BIU (beta=.220) and PE–>SAT–>BIU (beta=.227).

**Table 5:** Mediation effects (indirect effects).

Construct			Direct effects				Indirect effects				Specific paths		Specific effects			
			Beta value		*P* value		Beta value		*P* value				Beta value			*P* value
**SAT^a^**																
	PEOU^b^–>SAT	—^c^		—		.259^d^		.005		PEOU–>PE–>SAT		.259^d^			—	
**ATT^e^**																
	PE^f^–>ATT	.582^g^		<.001		.126		.19		PE–>SAT–>ATT		.126			—	
	PEOU–>ATT	.180		.11		.293^h^		.01		PEOU–>PE–>ATT		.241^h^			—	
	—	—		—		—		—		PEOU–>PE–>SAT–>ATT		.052			—	
**BIU^i^**																
	PE–>BIU	—		—		.495^g^		<.001		PE–>ATT–>BIU		.220^j^			.05	
	—	—		—		—		—		PE–>SAT–>BIU		.227^j^			.08	
	—	—		—		—		—		PE–>SAT–>ATT–>BIU		.048			—	
	PEOU–>BIU	—		—		.273^d^		.002		PEOU–>ATT–>BIU		.068			—	
	—	—		—		—		—		PEOU–>PE–>ATT–>BIU		.091			—	
	—	—		—		—		—		PEOU–>PE–>SAT–>BIU		.094			—	
	—	—		—		—		—		PEOU–>PE–>SAT–>ATT–>BIU		.020			—	
	SAT–>BIU	.362^j^		.07		.076		.31		SAT–>ATT–>BIU		.076			—	

^a^SAT: satisfaction.

^b^PEOU: perceived ease of use.

^c^Not applicable.

^d^*P*<.01.

^e^ATT: attitude.

^f^PE: perceived enjoyment.

^g^*P*<.001.

^h^*P*<.05.

^i^BIU: behavioral intention to use.

^j^*P*<.10.

## Discussion

### Key Findings and Theoretical Discussion

This study created a theoretical model to examine how determining factors from relevant theories of technology acceptance can be used to explain the acceptance of a novel VR-assisted MR training system created by our research team. The results suggested that ATT affected BIU (beta=.379; H2). This finding is consistent with the TAM [1]. We also found that PEOU’s effect on ATT was mediated by PE (beta=.241). The indirect effect of PEOU on BIU was mediated by other variables in the model (beta=.273). PEOU has been a crucial factor affecting users’ acceptance of information systems. In our study, it is likely that applying VR technology to both the hardware and software of the MR training system was relatively new and novel to the trainees. As most users were new to the VR system, they were not that familiar with its usage compared with other information technology they use in their everyday lives. Therefore, PEOU served as the predictive factor for their ATT toward and intention to use the VR-assisted MR training system. Whenever trainees perceived the use of our MR training system as easier, they had a better ATT toward the system and a higher intention to use it. By examining the key constructs’ direct and indirect effects, our study extended the current TAM theory from the context of information technology and systems to VR educational technology and further expanded the findings from recent applications of TAM in other VR-assisted systems (eg, [11,12]).

In addition, this study replicated the finding of the study conducted by Davis et al [13] that PE was affected by PEOU (beta=.414; H3). That is, trainees who regarded the MR training system as more effortless to use would perceive a higher level of enjoyment. In contrast, whenever users felt that using the system was challenging, their PE decreased. Furthermore, our study suggested that PE not only served as a variable that is influenced by PEOU but also served as the mediator between PEOU and other constructs in the model. Specifically, PE mediated the effect between PEOU and SAT (beta=.259) and partially mediated the effect of PEOU on ATT (beta=.241). Other than these findings, our study also verified that PE affected ATT (beta=.582; H4) and SAT (beta=.627; H5), as we had hypothesized. Those trainees who perceived a higher level of enjoyment had a more positive ATT toward and SAT with the system.

Extending the finding from the study by Davis et al [13] that PE played the predictive role in BIU and actual use, this study showed that PE affected BIU, mediated by ATT and SAT, specifically with the paths of PE–>SAT–>BIU (beta=.227) and PE–>ATT–>BIU (beta=.220). Users’ PE first affected their SAT with and ATT toward the system, which later influenced their intention to use the system. Our study, thus, extended Davies’ suggested psychological relationship between joyful perception and behavioral intention by adding 2 attitudinal factors, ATT and SAT, between them. In other words, a chain relationship, joyful perception–>attitudinal factors–>behavioral intention, was created. This study suggested the importance of ATT and SAT as the mediators when considering PE’s influence on BIU.

Finally, aligning with the ECM [16], we found that trainees’ SAT with the MR system directly predicted their intention to use it (beta=.362; H7). Whenever the VR-assisted training system satisfied the users, they were likely to adopt it for learning. SAT, thus, also played a mediating role between PE and BIU (beta=.227), as discussed in the previous paragraph.

### Practical Implications

On the basis of the findings of this established model, we also provide practical implications for MR trainers, technology developers, and education professionals. The empirical evidence of this study suggested that trainees’ intention to use the novel VR-assisted interactive MR training system was predicted by their PEOU and PE (indirectly via ATT or SAT). Given that the use of an MR training system enhances trainees’ MR ability [2], suggestions for technology developers and educators based on the previous 2 exogenous factors are discussed and aimed at helping relevant professionals better apply VR-assisted technologies and systems for education.

The predictive role of PEOU suggests that a user-friendly design is necessary for a VR-assisted MR training system. This requirement is understandable, as VR technology is relatively new and novel to consumers when compared with other information technology and systems on the market. VR technology developers are encouraged to keep improving system’s ease-of-use function, specifically with its hardware (eg, stereoscopic glasses and display and interactive controller), as well as the interactive design between the users and MR objects via the controller in the virtual environment. Future systems might want to consider eliminating tools and use of a physical controller and instead develop a motion sensor to completely rely on the trainees’ gestures or body movements to interact with the virtual content. These suggested approaches could effectively increase trainees’ PEOU, which would eventually lead to their better intention to use the training system.

PE, as we had predicted, served as the predictive factor for the use of the MR training system. We suggest that technology developers might further develop a system that complements the VR head-mounted display (HMD) that completely separates the real and virtual environments. An HMD could further create a better spatial presence by allowing users to actively switch their field-of-view rather than passively observe the content [33]. Previous studies found that whenever spatial presence increased, observers’ enjoyment amped up accordingly [34,35], as cited in the paper by Ravaja et al [36]. For the tasks of MR training, game scenarios such as stereoscopic 3D interactive Tetris could be created, which would likely be more playful for users. It is clearly supported by previous studies on digital learning (eg, [37,38]) that users in a game-playing situation perceived increased enjoyment.

### Limitations and Future Research

Similar to other studies, this research has its limitations and needs future research to further gauge unanswered questions. First, although all the causal hypotheses were based on established theories and previous studies, the nature of the data for our proposed theoretical model is cross-sectional. Future studies capable of conducting experiments are encouraged to investigate the relationships between the constructs explored in our model. Furthermore, this study explored the acceptance level of VR’s educational application in the context of MR. Other educational and training activities, which might apply VR technology in slightly different ways, are also worth further exploration. These extended studies would help researchers and practitioners have a holistic understanding regarding how VR technology can be accepted for educational purposes. Finally, although our proposed theoretical model explained nearly half (47.7%) of the variance of BIU, other VR-specific factors on trainees’ use of the novel VR-assisted interactive MR training system, such as perceived simulation and presence, need to be developed and incorporated into the model to better comprehend the remaining unanswered variance for adoption intention.

## Abbreviations

ATT: attitude
AVE: average variance extracted
BIU: behavioral intention to use
dof: degree-of-freedom
ECM: expectation-confirmation model
HMD: head-mounted display
MR: mental rotation
PE: perceived enjoyment
PEOU: perceived ease of use
PLS-SEM: partial least squares structural equation modeling
PU: perceived usefulness
SAT: satisfaction
SEM: structural equation modeling
TAM: technology acceptance model
VR: virtual reality
